# Xiaoqinglong Decoction Protects the Lungs of AECOPD Mice through the AMPK/mTOR Signaling Pathway

**DOI:** 10.1155/2020/9865290

**Published:** 2020-07-04

**Authors:** Qingsong Huang, Hongjing Yang, Chuantao Zhang, Jianying Wu, Wei Xiao, Zhu Zeng

**Affiliations:** ^1^Department of Respimiceory Medicine, Hospital of Chengdu University of Traditional Chinese Medicine, No. 39, Twelve Bridge Road, Jinniu District, Chengdu, Sichuan 610075, China; ^2^Department of Digestive Medicine, Hospital of Chengdu University of Traditional Chinese Medicine, Chengdu, Sichuan 610075, China

## Abstract

**Method:**

Male C57BL/6J mice were used to establish AECOPD model by cigarette smoke and bacterial exposure. Mice were randomly divided into normal control (NC), AECOPD, XQLD, Compound C (Com C), Com C + XQLD, and Clarithromycin (CLA) groups. After treatment, the pulmonary function was evaluated by whole-body plethysmograph. The lung histopathology was observed by HE staining. The serum levels of IL-6, TNF-*α*, and COX-2 were detected by ELISA assay. The apoptotic index was measured by TUNEL assay, and the protein expressions of Bax, Bcl-2, Caspase-3, GRP78, and CHOP in the lung tissues were measured by western blot assay.

**Results:**

XQLD treatment can improve pulmonary function (PF), ameliorate lung injury, and suppress inflammation and apoptosis of lung tissues. In addition, XQLD also markedly attenuated endoplasmic reticulum stress (ERS) and activated AMPK/mTOR pathway in the lung tissues of mice with AECOPD. However, the AMPK inhibitor Compound C decreased the protective effect of XQLD in AECOPD mice.

**Conclusion:**

These findings suggested that XQLD has protective effect against inflammation and apoptosis in AECOPD mice by attenuating ER stress via AMPK/mTOR pathway.

## 1. Introduction

COPD, a progressive inflammatory respiratory disease, is the fourth leading cause of morbidity and mortality worldwide and will rise to the third leading cause by 2030 [1]. COPD is characterized by presence of progressive and poorly reversible airflow limitation and linked to abnormal inflammatory response to harmful particles or gases in the airways and the lungs [2]. It is reported that multiple processes such as inflammation, cellular apoptosis, oxidative stress, and abnormal cellular repair are involved in the pathogenesis of COPD [3]. AECOPD is a serious event that is responsible for the progress of the disease and thus increased mortality [4]. Although drug treatments can control the established symptoms of AECOPD, no effective treatment available can delay or reverse the disease course [5]. In order to improve the survival of AECOPD patients, there is an urgent need to find a novel effective treatment to overcome AECOPD.

XQLD is a traditional Chinese medicine (TCM) including Gancao (*Glycyrrhizae Radix et Rhizoma*), Wuweizi (*Schisandra chinensis Fructus*), Ganjiang (*Zingiberis Rhizoma*), Guizhi (*Ramulus Cinnamomi*), Mahuang (*Ephedrae Herba*), Baishao (*Radix Paeoniae Alba*), Shaoyao (*Herbaceous peony*), Xixin (*Asari Radix et Rhizoma*), and Banxia (*Pinelliae Rhizoma*) [6]. XQLD has many pharmacological effects, such as antioxidation, anti-inflammation, and antiapoptosis, and is widely used against acute respiratory diseases in China [7]. However, few articles have studied the effectiveness of XQLD in treating inflammation and cellular apoptosis of lung in AECOPD. In the present study, XQLD was used to treat AECOPD mice model to observe the protective effect of XQLD on inflammation and apoptosis injury in AECOPD mice.

Adenosine monophosphate-activated protein kinase (AMPK) is a key sensor of cellular energy status, which has been reported to be involved in the development of COPD [8]. Qi et al. reported that the AMPK was inhibited in COPD rats [9]. In addition, Zhang et al. reported that the AMPK activators can prevent and halt COPD progression [10]. mTOR has been reported to suppress epithelial cell death and airway inflammation in cigarette smoke-induced COPD [11]. There is a close relationship between the signal cascade of mTOR and AMPK. miRNA-300/NAMPT inhibited inflammatory responses through activating AMPK/mTOR signaling pathway in neonatal sepsis [12]. The endoplasmic reticulum (ER) is central to many cellular functions and is responsible for protein folding and biosynthesis. Endoplasmic reticulum stress is due to the imbalance of unfolded or misfolded proteins caused by free radicals and hypoxia in endoplasmic reticulum lumen, which leads to the apoptosis of bronchial and alveolar epithelial cells. Meanwhile, a previous study showed that AMPK agonist AICAR can reduce endoplasmic reticulum stress, thereby reducing inflammation and apoptosis in COPD mice [9]. Therefore, the aim of this study was to investigate whether XQLD protects against COPD by attenuating apoptosis and ER stress through AMPK/mTOR signaling pathway in mice.

## 2. Materials and Methods

### 2.1. XQLD Preparation

XQLD was prepared according to the method reported by Song et al. [7, 13]. Briefly, 14 g of *Ephedra*, 16 g of *Cassia twig*, 36 g of *Glycyrrhizae Radix*, 18 g of *Asarum*, 18 g of *Schisandra chinensis*, 18 g of *Zingiberis Rhizoma*, 54 g of *Pinellia ternata*, and 14 g of *Radix Paeoniae Alba* were bought from the pharmacy of the Hospital of Chengdu University of TCM (Chengdu, China). These herbs were macerated in 1600 mL of water for 3 h at room temperature. Then the mixture was boiled until the total volume reduced to 500 mL. All extracts then were stored at 4°C.

### 2.2. Experimental Animals and Grouping

Eight-week-old male C57BL/6J mice (20 ± 2 g) were purchased from Chengdu Dossy Experimental Animals Co. Ltd. (Sichuan, China), then were maintained in animal room in 12 h light-dark cycle, and allowed free access to water and standard diet. After one week of conditioning, 48 mice were randomly assigned to 6 groups (*n* = 8 per group): normal control group (NC), AECOPD, XQLD, Compound C group (Com C), XQLD + Compound C group (XQLD + Com), and Clarithromycin group (CLA). All the groups were AECOPD model mice except the normal control group. The experimental protocol for the care and use of laboratory animals was approved by the Experimental Animal Ethics Committee of West China Hospital of Sichuan University (Chengdu, China). The associated permit number is 2019167A.

### 2.3. Model Establishment

The detailed procedures for AECOPD in mice model induced by combined cigarette smoke with *Klebsiella pneumoniae* were described in a previous report [14]. Briefly, the mice were placed in a chamber (70 cm × 50 cm × 30 cm) with smoke produced by the combustion of tobacco (Sichuan, China; tar 10 mg, carbon monoxide 12 mg, and nicotine 0.48 mg) for 30 min, twice a day for 8 weeks. To mimic acute exacerbation, the mice were subjected to nasal inhalation of 0.1 mL of 6 × 10^8^ CFU/mL *Klebsiella pneumoniae* solution, once every 5 days for 8 weeks (strain: 46114) to establish the mice models.

### 2.4. Drug Treatment

The drug treatments were initiated on day 57. The mice in NC and AECOPD groups were given 0.9% normal saline by gavage. The mice in XQLD and Com C groups were given Xiaoqinglong decoction (7.5 g/kg) and Compound C (10 mg/kg) by gavage for 14 d, respectively. The mice in XQLD + Com C group were given Xiaoqinglong decoction (7.5 g/kg) together with Compound C (10 mg/kg) by gavage for 14 d. The mice in CLA group were given Clarithromycin (70 mg/kg) by gavage for 14 d, as a positive control group.

### 2.5. Pulmonary Function

The lung function of mice was detected by the animal pulmonary functionality test machine AniRes 2005 (Bestlab, Beijing, China) after the last exposure. Briefly, mice were placed in a sealed box that was connected to transducers and a computer and allowed to acclimate for 5 min. Afterwards, pulmonary function, including peak expiratory flow (PEF), peak inspiratory flow (PIF), and minute volume (MV), was assessed.

### 2.6. Cytokine Analysis

Immediately after pulmonary function measurement, the blood was collected and centrifuged at 5000 × g at 4°C for 10 min. Then the serum was stored at −80°C. IL-6, COX-2, and TNF-*α* serum levels were measured by ELISA kit (MultiSciences, Hangzhou, China) according to the manufacturer's instructions.

### 2.7. HE Staining and TUNEL Staining

The right lobe of the lung was removed and fixed with 4% paraformaldehyde for 24 h, dehydrated, and embedded in paraffin. Then the tissues were cut into 5 *µ*m thick sections that were stained with hematoxylin and eosin (HE) and observed by light microscope.

The apoptosis in the lung tissues was detected using a TUNEL assay kit (Sangon Biotech, Shanghai, China) according to the manufacturer's instructions. The apoptotic cells exhibited brown staining within the nucleus. Images were captured with a fluorescence microscope (Olympus Corporation, Tokyo, Japan). The images of tunnel staining were analyzed by Image-Pro Plus software.

### 2.8. Western Blot Assay

The lung tissues were homogenized with RIPA lysis buffer (Boster, Wuhan, China), and the protein concentration was quantified with a protein assay kit (Wuhan Boster Biological Technology, Ltd., Wuhan, China). An equal amount of protein in each group was separated by 10% sulphate-polyacrylamide gel electrophoresis (SDS-PAGE) and then transferred onto a PVDF membrane (Millipore, MA, USA). The membranes were sealed with 5% nonfat milk powder at room temperature for 1 h and then were incubated with rabbit monoclonal antibody against CHOP (Abcam, USA, dilution 1 : 500), GRP78 (Abcam, USA, dilution 1 : 1,000), Caspase-3 (Abcam, USA, dilution 1 : 500), Bcl-2 (Abcam, USA, dilution 1 : 1,000), Bax (Abcam, USA, dilution 1 : 1,000), AMPK (Abcam, USA, dilution 1 : 500), p-AMPK (Abcam, USA, dilution 1 : 500), mTOR (Abcam, USA, dilution 1 : 500), p-mTOR (Abcam, USA, dilution 1 : 500), and *β*-actin (Abcam, USA, dilution 1 : 2,000) at 4°C overnight. Following this, the membrane was washed with PBS at room temperature and then incubated with secondary antibody of goat anti-rabbit immunoglobulin G (IgG) (Abcam, USA, 1 : 2,000) at room temperature for 1 h. At last, protein bands were visualized using an ECL chemiluminescence kit (EMD Millipore, USA) and assayed by quantitative image analysis software. *β*-actin was used as the internal reference.

### 2.9. Statistical Analysis

The SPSS 19.0 statistical software was used to perform the statistical analyses, Student's *t*-test was used to analyze two groups, and one-way analysis of variance (ANOVA) was used to examine multiple groups. *P* < 0.05 was considered statistically significant. The data were expressed as mean value ± standard deviation.

## 3. Results

### 3.1. XQLD Improved the Pulmonary Function in AECOPD Mice

**Figure 1:**
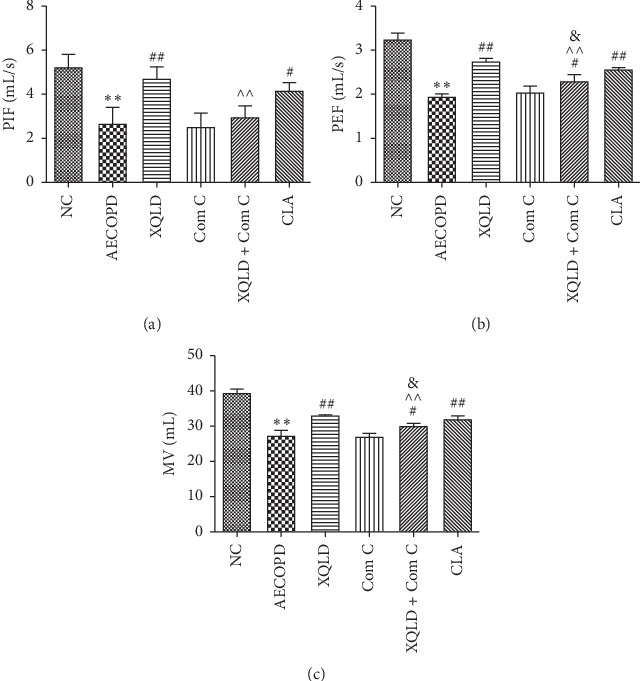
The effect of XQLD on pulmonary function in the AECOPD mice. (a) Peak inspiratory flow (PIF). (b) Peak expiratory flow (PEF). (c) Minute volume (MV). Data are mean ± SD (*n* = 8). NC: normal control; XQLD : Xiaoqinglong decoction; Com C: Compound C; CLA : Clarithromycin. ^*∗∗*^*P* < 0.01 compared with the NC group, ^#^*P* < 0.05, ^##^*P* < 0.01 compared with the AECOPD group. ^^^^*P* < 0.01 compared with the XQLD group, ^&^*P* < 0.01 compared with the Com C group.

Lung function test showed that PEF, PIF, and MV were significantly lower in the AECOPD group than those in the NC group (*P* < 0.01). Compared with the AECOPD group, the PEF, PIF, and MV were significantly higher in the XQLD and CLA groups (*P* < 0.01 or *P* < 0.05). Compared with the Com C group, the PEF and MV in XQLD + Com C group were significantly increased (*P* < 0.05). Meanwhile, the PEF, PIF, and MV were significantly lower in the XQLD + Com C group than in the XQLD group (*P* < 0.01) (Figure 1).

### 3.2. XQLD Reduced Pulmonary Histopathological Injury in AECOPD Mice

**Figure 2:**
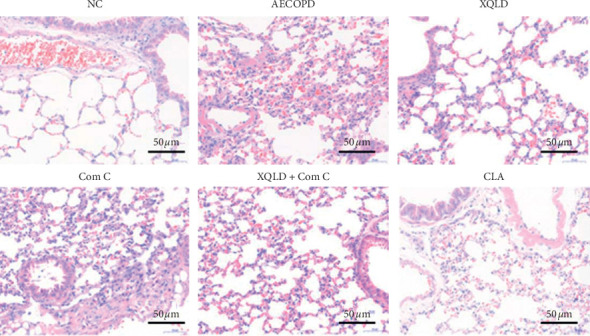
XQLD improved the degree of lung parenchyma injury in AECOPD mice. Lung tissue was stained using HE (×200). NC: normal control; XQLD : Xiaoqinglong decoction; Com C: Compound C; CLA : Clarithromycin.

As shown in Figure 2, compared with the NC groups, bronchial epithelial cell exfoliation, nuclear fragmentation of epithelium, pulmonary arterial wall thickening, alveolar break, fusion, and inflammatory cell infiltration were observed in the AECOPD and Com C groups. Xiaoqinglong decoction and Clarithromycin can help to relieve the typical pathological features of AECOPD. In the XQLD and CLA groups, inflammatory cell infiltration and destruction of alveolar septa were reduced. In addition, the alveolar septa retained a relatively normal structure. Compared with the AECOPD groups, inflammatory cell infiltration and nuclear fragmentation of epithelium were slightly reduced in the XQLD + Com C group, but obstructive emphysema and some alveolar break and fusion were observed.

### 3.3. XQLD Reduced Inflammatory Cytokines in AECOPD Mice

**Figure 3:**
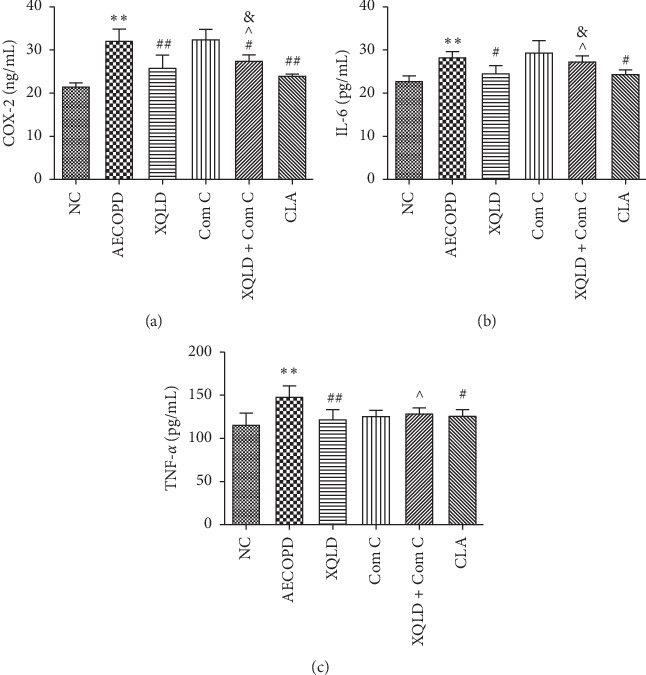
XQLD reduced the AECOPD-mediated release of inflammatory cytokines. COX-2, IL-6, and TNF-*α* were measured by ELISA assay. (a) The content of COX-2 in each group. (b) The content of IL-6 in each group. (c) The content of TNF-*α* in each group. Data are mean ± SD (*n* = 8). NC: normal control; XQLD : Xiaoqinglong decoction; Com C: Compound C; CLA : Clarithromycin. ^*∗∗*^*P* < 0.01 compared with the NC group, ^#^*P* < 0.05, ^##^*P* < 0.01 compared with the AECOPD group. ^^^*P* < 0.05 compared with the XQLD group, ^&^*P* < 0.01 compared with the Com C group.

As shown in Figure 3, compared to the NC groups, the serum levels of COX-2, IL-6, and TNF-*α* in the AECOPD group significantly increased (*P* < 0.01). Compared with the AECOPD group, the serum levels of COX-2, IL-6, and TNF-*α* in the XQLD and CLA groups were significantly reduced (*P* < 0.05 or *P* < 0.01). Compared with Com C group, the serum levels of COX-2 and IL-6 in the XQLD + Com C group were significantly decreased (*P* < 0.05). In addition, the serum levels of COX-2, IL-6, and TNF-*α* in the XQLD + Com C group were significantly increased (*P* < 0.05) compared with the XQLD groups.

### 3.4. XQLD Suppressed Pulmonary Apoptosis in Mice AECOPD Model through Downregulation of the AMPK/mTOR Signaling Pathway

**Figure 4:**
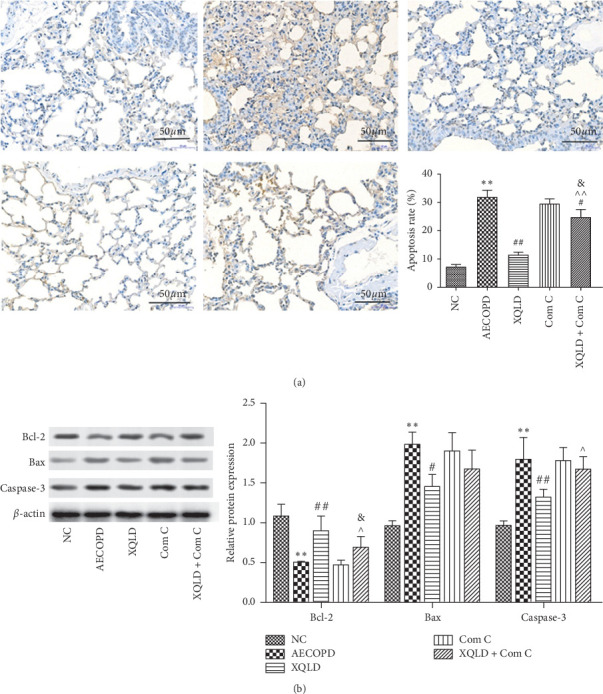
XQLD reduced the AECOPD-mediated cell apoptosis in mice. (a) Images of lung tissue following TUNEL staining; nucleus of apoptotic cells were stained brown. (b) Bcl-2, Bax, and Caspase-3 expression levels were measured by western blot assay. Data were expressed as mean ± SD (*n* = 8). NC: normal control; XQLD : Xiaoqinglong decoction; Com C : Compound C; CLA : Clarithromycin. ^*∗∗*^*P* < 0.01 compared with the NC group; ^#^*P* < 0.05, ^##^*P* < 0.01 compared with the AECOPD group; ^*P* < 0.05, ^^^^*P* < 0.01 compared with the XQLD group; ^&^*P* < 0.01 compared with the Com C group.

The results of the TUNEL assay are shown in Figure 4 A. Compared with the NC group, the TUNEL-positive cell number in the AECOPD group was significantly increased (*P* < 0.01). In comparison to the AECOPD group, the TUNEL-positive cell number in XQLD and XQLD + Com C groups of mice was significantly reduced (*P* < 0.05 or *P* < 0.01). Compared with Com C group, the TUNEL-positive cell number in the XQLD + Com C group was significantly decreased (*P* < 0.05). Meanwhile, compared with XQLD group, the cell apoptosis was significantly increased in XQLD + Com C group (*P* < 0.01).

To further characterize the effects of XQLD against apoptosis in AECOPD, we analyzed the protein expression of Bcl-2, Bax, and Caspase-3 in the lung tissues of various groups. As shown in Figure 4(b), the protein expression of Bax and Caspase-3 was significantly increased in the AECOPD group compared with NC group, but Bcl-2 was significantly decreased (*P* < 0.01). The levels of Bax and Caspase-3 protein were significantly lower in the XQLD group than those in the AECOPD group, while the level of Bcl-2 was increased significantly (*P* < 0.01). Compared with Com C group, the levels of Bcl-2 in the XQLD + Com C group were significantly increased (*P* < 0.05). Compared with XQLD group, the protein level of Caspase-3 in XQLD + Com C group increased significantly (*P* < 0.05). However, the level of Bcl-2 decreased significantly (*P* < 0.05). There was no significant difference in Bax protein between XQLD group and XQLD + Com C group.

### 3.5. Effect of XQLD on the AMPK/mTOR Signaling Pathway in the Lung Tissues of Mice with AECOPD

**Figure 5:**
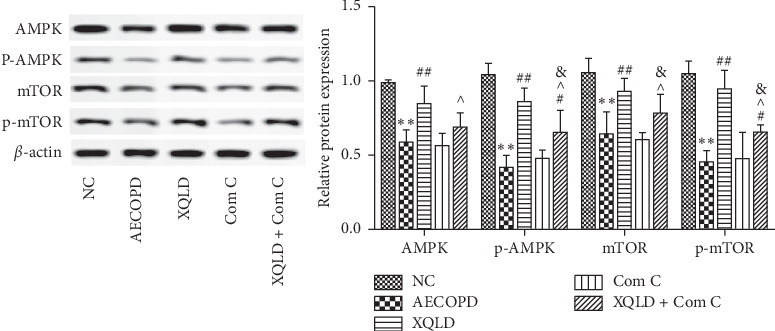
XQLD activated the AMPK/mTOR signaling pathway. The protein expression of AMPK, p-AMPK, mTOR, and p-mTOR was measured by western blot. Data were expressed as mean ± SD (*n* = 8). NC: normal control; XQLD : Xiaoqinglong decoction; Com C : Compound C. ^*∗∗*^*P* < 0.01 compared with the NC group; ^#^*P* < 0.05, ^##^*P* < 0.01 compared with the AECOPD group; ^^^*P* < 0.05 compared with the XQLD group; ^&^*P* < 0.01 compared with the Com C group.

AMPK is regarded as a therapeutic target for COPD. To investigate whether XQLD activates AMPK/mTOR pathway in COPD, we measured the protein expression of AMPK, mTOR, p-AMPK, and p-mTOR in the lung tissues of various groups (Figure 5). The results of western blot analysis indicated that the protein expression of AMPK, p-AMPK, mTOR, and p-mTOR was significantly decreased in the AECOPD group as compared with the NC group (*P* < 0.01). However, treatment with XQLD significantly increased the protein expressions of AMPK, p-AMPK, mTOR, and p-mTOR as compared with the AECOPD group (*P* < 0.01). Compared with the Com C group, the protein expression of p-AMPK, mTOR, and p-mTOR significantly increased in XQLD + Com C group (*P* < 0.05). In addition, compared with the XQLD group, the protein expression of AMPK, p-AMPK, mTOR, and p-mTOR significantly decreased in XQLD + Com C group (*P* < 0.05).

### 3.6. Effect of XQLD on ER Stress in the Lung Tissues of Mice with AECOPD

**Figure 6:**
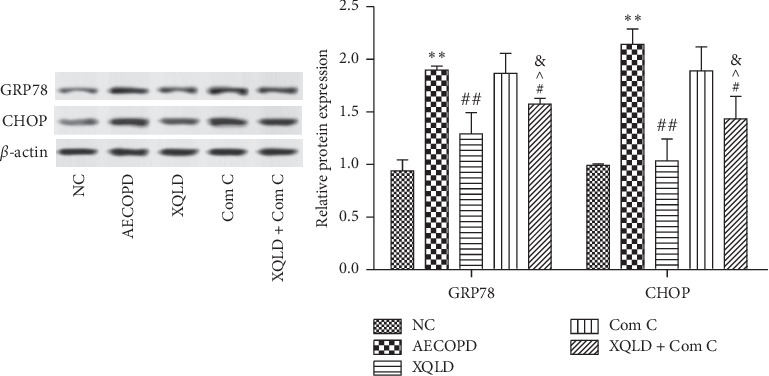
XQLD suppressed ER stress in the lung tissues of mice with COPD via the AMPK/mTOR signaling pathway. The protein expression of GRP78 and CHOP was measured by western blot. Data were expressed as mean ± SD (*n* = 8). NC: normal control; XQLD : Xiaoqinglong decoction; Com C: Compound C ^*∗∗*^*P* < 0.01 compared with the NC group; ^#^*P* < 0.05, ^##^*P* < 0.01 compared with the AECOPD group; ^^^*P* < 0.05 compared with the XQLD group; ^&^*P* < 0.01 compared with the Com C group.

The ER stress-mediated apoptosis is involved in the development of COPD. To analyze the effect of XQLD on ER stress in AECOPD, we analyzed the protein expressions of CHOP and GRP78 in the lung tissues of mice. As shown in Figure 6, compared with NC group, the protein expression of CHOP and GRP78 was significantly increased in the AECOPD group. Compared with the AECOPD group, the protein expressions of CHOP and GRP78 were significantly decreased in the XQLD and XQLD + Com C group (*P* < 0.05 or *P* < 0.01). Compared with the Com C group, the protein expressions of CHOP and GRP78 were significantly decreased in the XQLD and XQLD + Com C groups (*P* < 0.05). Meanwhile, the AMPK inhibitor Compound C significantly reduced the effects exerted by XQLD, which caused significant differences between XQLD and XQLD + Com C groups (*P* < 0.05).

## 4. Discussion

Exacerbation of COPD is recognized as a major event in the natural history of COPD, because repeated exacerbation has negative effect on survival period of COPD patients. Previous studies have demonstrated that inflammation response and apoptosis of bronchial and alveolar epithelial cells play a critical role in the development of COPD. In COPD patients, the levels of several inflammatory mediators such as IFN-*γ*, IL-1*β*, IL-13, COX-2, IL-6, and TNF-*α* in serum from COPD patients are increased [15–17]. Several studies have demonstrated that the apoptosis in the lung tissues resulted in small airway abnormality and parenchymal destruction in COPD [18, 19]. However, there are no effective therapies for inflammation and apoptosis in the lung tissues of AECOPD. Although macrolide antibiotics can suppress bacterial infection and inflammation in COPD patients, their use is limited by bacterial resistance and long-term use that leads to hearing loss and cardiovascular disease. XQLD, a classical prescription designed based on the TCM theory and clinical experience, proved to be capable of warming the lungs, dissipating excessive fluid, and relieving cough and asthma [13]. It is widely used for the treatment of asthma triggered by upper respiratory infections [20]. A previous clinical study showed that XQLD administration significantly improved AECOPD patients' symptoms [21]. However, there remains a lack of researches on the protective effect of XQLD on COPD, and the underlying mechanisms of XQLD remain unclear.

COPD is characterized by chronic airflow limitation, measured spirometrically by the ratio of the forced expiratory volume (FEV) to the forced vital capacity (FVC) and minute ventilation volume (MV) [22]. In COPD patients, the lung function sharply declines during the disease exacerbation [22]. Inflammatory factors are involved in the progressive development of airflow limitation and structural damage of airway wall and lung parenchyma [23]. Therefore, lung function and inflammatory factors are widely used as representative biomarkers reflecting the occurrence and status of exacerbation of AECOPD. Specifically, in this study, it was demonstrated that the administration of XQLD could significantly improve the PEF, PIF, and MV. Meanwhile, XQLD decreased the levels of proinflammatory cytokines including IL-6, COX-2, and TNF-*α* in the COPD mice. In addition, it was demonstrated that XQLD suppressed lung parenchyma destruction and inflammatory cells infiltration, and the effect is consistent with the Clarithromycin. TUNEL staining showed that XQLD could significantly ameliorate the bronchial and alveolar epithelial cell apoptosis rate in lung tissues compared with the AECOPD group. In addition, western blot analysis showed that XQLD significantly decreased the expression of the proapoptotic Bax and Caspase-3 protein, while increasing the antiapoptotic Bcl-2 protein. Therefore, our results indicated that XQLD can reduce the risk of COPD exacerbations via inhibiting proinflammatory expression and ameliorating apoptosis in the lung tissues of mice with AECOPD.

Studies have confirmed that ERS is a potential pathological mechanism for COPD and lung parenchyma destruction. Kelsen et al. [24] reported that ER stress is activated by cigarette smoke in lung tissues of patients with COPD. He et al. [25] reported that inhibiting ER stress exerted protective effects against COPD by inhibiting the apoptosis of bronchial and alveolar epithelial cells. Moreover, Zhao et al. [26] found that ER stress played an important role in the apoptosis of alveolar epithelial apoptosis in COPD, and adiponectin can ameliorate the progression of COPD through inhibiting the ERS-induced alveolar epithelial apoptosis in rats. Therefore, inhibition of ER stress-mediated apoptosis is a key therapeutic approach for COPD. Some studies have confirmed that ERS related proteins GRP78 and CHOP can mediate apoptosis through caspase-3 pathways [27]. In the present study, we found that expression of ERS related proteins GRP78 and CHOP in the lung tissues of the AECOPD group was increased as compared with the NC group, indicating that AECOPD could induce ERS. Meanwhile, we demonstrated that the administration of XQLD significantly decreased protein levels of GRP78 and CHOP as compared with the AECOPD group, which indicated that XQLD protected against bronchial and alveolar epithelial cell apoptosis maybe by inhibiting ER stress in COPD.

Studies have shown that the AMPK signaling pathway is involved in the development of AECOPD. Lee et al. found that cigarette smoke induced more extensive lung inflammation and emphysema in AMPK*α*1-deficient mice [28]. Wang et al. [29] reported that AMPK is activated and mTOR is suppressed by 12 h PM 2.5 exposure in human lung cancer epithelial cells. In this study, the data demonstrated that AMPK, mTOR, p-AMPK, and p-mTOR protein decreased markedly in AECOPD mice compared with normal mice and notably increased after XQLD treatment. More importantly, a previous study has shown that exercise training induced Akt/mTOR pathway activation in COPD patients [30]. Taken together, the observation indicated that XQLD could activate the AMPK/mTOR pathway in AECOPD mice.

The AMPK/mTOR signaling pathway plays an essential role in limiting acute inflammatory reactions and apoptosis via suppressing ER stress. However, whether XQLD ameliorates inflammatory and apoptosis through AMPK/mTOR signaling pathway in COPD remains unclear. In the present study, we found that XQLD inhibited ERS in lung tissues of AECOPD mice, while Compound C (AMPK inhibitor) upregulated ER stress related proteins CHOP and GRP78 in the lung tissues as compared with the XQLD group. Meanwhile, Compound C treatment decreased the protective effect of XQLD on the pulmonary function, inflammation, and apoptosis of lung tissues in AECOPD mice. Together, our study indicated that XQLD improved pulmonary function, alleviated lung injury, and inhibited ER-stress-induced cell apoptosis and inflammation via activation of the AMPK/mTOR pathway.

In conclusion, as a kind of traditional Chinese medicine, XQLD can attenuate inflammation and apoptosis of lung tissues and ameliorate pulmonary function after AECOPD. What is more, the protective effect of XQLD was attributed to attenuating ERS in AECOPD via upregulating AMPK/mTOR pathway. This finding might have clinical value for the treatment of AECOPD.

## Data Availability

The data used or analyzed during the current study are available from the corresponding author on reasonable request.
